# Short‐Term Exposure to Ambient Air Pollution and Hospitalization Risks and Costs on Type 2 Diabetes With Three Typical Comorbidities in Sichuan, China: A Time‐Stratified Case‐Crossover Study

**DOI:** 10.1029/2025GH001629

**Published:** 2026-05-25

**Authors:** Wanyanhan Jiang, Han Chen, Yuelin Zhou, Hongwei Li, Lulu Lian, Lian Yang

**Affiliations:** ^1^ School of Public Health Chengdu University of Traditional Chinese Medicine Chengdu China; ^2^ Sichuan Wanhao Consulting Co., Ltd Chengdu China; ^3^ School of Management Chengdu University of Traditional Chinese Medicine Chengdu China; ^4^ Center for Earth System Modeling Research Lanzhou University Lanzhou China

**Keywords:** air pollution, short‐term, hospital admissions, attributable economic cost, type 2 diabetes with comorbidities

## Abstract

This study is aimed at examining the associations and hospitalization costs between air pollutants and hospital admissions (HAs) for type 2 diabetes mellitus (T2DM) with three typical comorbidities, namely cardiovascular disease (CVD), hypertension, and peripheral vascular disease (PVD). Conditional Poisson regression based on Poisson distribution was used to confirm the relationship between air pollution and HAs for T2DM with comorbidities. Potential effect modifiers, such as age and gender, were also involved. The attributable hospitalization number and costs due to air pollutants exposure were estimated, using WHO's air quality guidelines as reference. In total, 92,381 T2DM HAs were recorded, of which 6,855 patients with CVD, 23,403 with hypertension, and 21,207 with PVD, respectively. The significant short‐term effects of NO_2_, PM_10_, PM_2.5_ and SO_2_ on HAs for T2DM with CVD, hypertension and PVD were different, mainly concentrated on lag 6 and lag 7. Stratified analysis demonstrated that mainly female and elder people (≥45 years) were more vulnerable to air pollutants. Total hospitalization costs attributable to air pollution were substantial. For T2DM patients with CVD, the hospitalization costs linked to NO_2_, PM_10_, and PM_2.5_ were 7.43, 3.04, and 6.79 million China Yuan (CNY), respectively. For the hypertension comorbidity group, costs reached 12.17 million CNY for NO_2_ and 10.89 million CNY for PM_10_. Additionally, NO_2_ exposure for 10.82 million CNY in hospitalization costs for T2DM patients with PVD. These findings underscore that air pollution not only exacerbates health risks but also imposes a significant financial strain on the healthcare system for vulnerable populations.

## Plain Language Summary

We investigated how short‐term exposure to air pollution affects hospitalizations and costs for people with type 2 diabetes (T2DM) and with three common comorbidities: cardiovascular disease, hypertension, or peripheral vascular disease (PVD). Using conditional Poisson regression based on Poisson distribution, we found that air pollution significantly increases the risk of hospitalization for these vulnerable patients. For individuals with T2DM and also with cardiovascular disease, hypertension, or PVD, exposure to air pollutants like NO_2_, PM_10_, PM_2.5_, and SO_2_ significantly increased their risk of hospitalization. These effects were most pronounced 6–7 days after exposure. Our analysis showed female and older adults (≥45 years) are more susceptible to the negative effects of air pollution. We also calculated the financial burden. The total hospitalization costs attributable to air pollution for these patients amounted to tens of millions of Chinese Yuan (CNY). This suggests that improving air quality is not only crucial for protecting the health of people with T2DM and these comorbidities, but also for reducing a major healthcare cost. Future public health efforts should focus on these particularly susceptible groups.

## Key Points

- The short‐term effect of air pollution and disease burden of type 2 diabetes mellitus with three typical comorbidities in Sichuan, China

## 1 Introduction

Type 2 diabetes mellitus (T2DM) is one of the most common and costly chronic diseases, with the prevalence increasing for decades worldwide. The global prevalence of diabetes was estimated to be 9.3% (463 million people) in 2019 and is rising (Saeedi et al., 2019). According to the International Diabetes Federation, there are 537 million people suffering from diabetes in 2021, mainly concentrated in Asia with China as one of the most epidemic countries. And the number is predicted to increase to 783 million in 2045. Particularly, at least US$966 billion costs to global health economies were estimated, putting economic pressure on diabetes patients and society (Sun et al., 2022). T2DM is complicated, since the link with multiple comorbidities, like cardiovascular disease (CVD), hypertension, and peripheral vascular disease (PVD). These comorbidities would increase mortality and morbidity, rendering significantly high economic burden to T2DM patients. In real world, T2DM is always associated with comorbidity and rarely involved in isolation, for those with comorbidities would spend up to three times as much as those without comorbidities (Azami & Ebrahimy, 2023).

In recent decades, owing to the acceleration of industrialization and urbanization, substantial air pollution has been discharged into environment, resulting in noticeable public health problems. Fine particulate matter (PM) from sources such as industrial production and automobile engines is easily inhaled and widely dispersed throughout the body, often carrying toxic and harmful substances. As a result, air pollution would affect nearly every organ, introducing many diseases, such as CVD, hypertension and even death. According to the Global Burden of Disease study, air pollution is the fourth leading risk factor for human mortality, which causes 6.67 million deaths worldwide in 2019 (Murray et al., 2020).

Recently, epidemiology studies of air pollution and chronic disease are increasing sharply. Short‐term exposure to air pollution would be a risk factor has been found contributing to hospital admissions (HAs) and costs for T2DM (C. Liu et al., 2013, X. Liu et al., 2021; Rajagopalan & Brook, 2012). In a study conducted in Chile, it reported that an interquartile range increase in nitrogen dioxide (NO_2_), PM with aerodynamic diameter ≤2.5 μm (PM_2.5_), ≤10 μm (PM_10_) and sulfur dioxide (SO_2_) were involved with relative risks of hospitalization for diabetes as 1.12 (95% confidence interval (CI): 1.05, 1.20), 1.11 (95% CI: 1.06, 1.16), 1.11 (95% CI: 1.07, 1.15), 1.14 (95% CI: 1.06, 1.22), separately (Dales et al., 2012). There are 3.2 million cases of diabetes patients, with 8.3 million decreasing disability‐adjusted life years globally, owing to the involvement with air PM (Benjamin et al., 2018). Besides, air pollution is a crucial risk of CVD, hypertension and PVD, presenting positive relationships with these three typical comorbidities of diabetes (Cai et al., 2016; Jiang et al., 2022; Xin et al., 2023). Thus, when exposure to air pollution, the CVD, hypertension and PVD patients with T2DM would associate with hospitalization risks, which have largely remained unknown.

China has the most T2DM people in the world, with 140.9 million Chinese adults in 2021 (Sun et al., 2022). When compared with people without diabetes, T2DM patients had a 15% increased risk of all‐cause mortality (Tancredi et al., 2015). In China, T2DM and the linking comorbidities has become a central public health issue since the associated hospitalization cost. Until now, limited studies concerning on T2DM patients with comorbidities were implemented in China, even though the prevalence of diabetes increased markedly from 10.9% in 2013 to 12.4% in 2018 in China (Wang et al., 2021). And there are gaps for the hospitalization costs of diabetic comorbidities in areas with high air pollution, like Sichuan Province, which was the fourth high air concentrations in China (Xiong et al., 2019). Situated in the Sichuan Basin, not only with unfavorable geographical conditions, but also the high industrialization and population densities would render heavy air pollution (Q. Zhang et al., 2009). Furthermore, the residents with diabetes in Sichuan Province accounted for 6%–7% (about 8.6 million individuals) of the national population with diabetes (Wu et al., 2022). Nevertheless, studies regarding short‐term effect of ambient air pollution on HAs for T2DM patients with comorbidities are rare in China, not to speak of related hospitalization costs.

The purpose of the present study is to evaluate the relationships between air pollutants and diabetes patients with three typical comorbidities (CVD, hypertension and PVD) and hospitalization costs in nine cities/prefectures in Sichuan Province during 2017–2019, to provide scientific evidence for controlling and reducing diabetes in China.

## 2 Materials and Methods

### 2.1 Study Area

**Figure 1:**
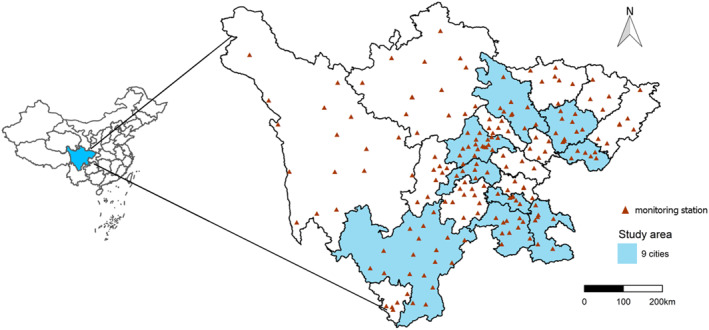
Geographical distribution of study areas, nine cities/prefectures of Sichuan Province.

Located in southwest China, Sichuan Province consists of the Sichuan Basin and West Sichuan Plateau. There are 21 cities/prefectures, with 18 cities in the Sichuan Basin and 3 autonomous prefectures in the West Sichuan Plateau. The study area of this study was the 9 cities/prefectures in Sichuan Province, Chengdu (CD), Mianyang (MY), Nanchong (NC), Guang'an (GA), Meishan (MS), Zigong (ZG), Yibin (YB), Luzhou (LZ), and Liangshanzhou Yi Autonomous Prefecture (LSZ), among them 8 from Sichuan Basin and 1 from the West Sichuan Plateau (Figure 1). As shown, the study area involves various economies and population to some extent.

### 2.2 Hospitalization Data

The hospitalization data were obtained from the hospital electronic health records of 139 tertiary and secondary, with the exceptions of Luzhou and Nanchong, each of which included only one provincial hospital. For each county, at least one Western Medicine Hospital, one Traditional Chinese Medicine Hospital, and one Maternal and Child Health Hospital were included. Additionally, some tertiary and secondary hospitals were included to further ensure representativeness. The distribution of these hospitals across regions was as follows: 38 in Chengdu, 25 in Mianyang, 13 in Guang'an, 13 in Meishan, 22 in Zigong, 15 in Yibin, and 11 in Liangshan Prefecture. The hospitalization data were collected from hospital electronic health records in these 9 cities/prefectures during 1 January 2017, to 31 December 2019.

The data included basic demographics, dates of admission and discharge, hospitalization costs, primary disease diagnosis, and disease code based on the International Classification of Disease, 10th Revision (ICD‐10). A small proportion of records (approximately 5%) with ambiguous entries, typos, or informal markings were manually coded by a trained researcher following the official ICD‐10 guidelines. To ensure data integrity, a random sample of these manually coded entries was cross‐checked by a second independent researcher, achieving a high level of inter‐rater reliability. T2DM patients with comorbidities were selected in two steps. First, hospital admissions with a primary diagnosis of T2DM were identified using the ICD‐10 code E11. Second, among these admissions, comorbidities were identified from all diagnosis fields using ICD‐10 codes I20–I25 for CVD, I10 for hypertension, and I73.9 for PVD. It is important to note that our selection strategy restricted the study population to hospitalizations with T2DM as the primary diagnosis. Therefore, our findings may not be generalizable to patients with T2DM who were hospitalized primarily because of a severe comorbid event.

### 2.3 Air Pollution and Meteorological Data

Ambient daily air pollution data of NO_2_, PM_10_, PM_2.5_, and SO_2_ were obtained from Sichuan Ecological Environmental Monitoring Station from 2017 to 2019. There are 183 monitoring stations in Sichuan Province. Sichuan Province has 183 provincial air pollution monitoring stations, distributed as follows: 19 in Chengdu, 9 in Mianyang, 6 in Guang'an, 6 in Meishan, 6 in Zigong, 10 in Yibin, 17 in Liangshan Prefecture, 13 in Aba Prefecture, 18 in Ganzi Prefecture, 6 in Bazhong, 7 in Dazhou, 6 in Deyang, 7 in Guangyuan, 11 in Leshan, 7 in Luzhou, 6 in Meishan, 9 in Nanchong, 5 in Neijiang, 5 in Panzhihua, 5 in Suining, 8 in Ya'an, and 3 in Ziyang. Given that the study population originates from across the entire province, data from all provincial air pollution monitoring stations were utilized. The mean levels of NO_2_, PM_10_, PM_2.5_, and SO_2_ were 30.0, 71.7, 46.0, and 12.5 μg/m^3^, and the mean temperature and relative humidity are 17.4°C and 77.2%, respectively. Meteorological data were collected from Sichuan Meteorological Bureau during the same period.

Inverse distance weighting (IDW) method was adopted to solve the missing data, which was a common and typical spatial interpolation method in environmental studies (Byun & Schere, 2006). Based on the weighted average, IDW interpolation was calculated from the point source data like all provincial environmental and meteorological stations in this study (Shepard, 1968; Su et al., 2018). In addition, the AutoNavi Maps API (https://lbs.amap.com/) was involved to geocode the home addresses of all T2DM cases and monitoring stations. The air pollution exposure for each case was then estimated as the inverse distance squared (1/d^2^) weighted average of concentrations measured at all monitoring stations.

### 2.4 Modeling

#### 2.4.1 Statistical Methods

The time‐series case‐crossover design with conditional Poisson regression was adopted to confirm the relationship between air pollutants and hospitalized T2DM patients with comorbidities, with temperature and relative humidity as confounding factors. Since daily T2DM cases were low‐probability and quantitative event, Poisson distribution was involved. Control and case days are only compared if they are in the same stratum. To remove any confounding by day of the week, control days are matched to case days if they are on the same day of the week. Based on case‐crossover design framework, the influence of long‐term tendency, seasonal effect, and day of week could be adjusted (Zanobetti et al., 2009). The Relative Risk (RR) and 95% CI were calculated for per 1 μg/m^3^ increase of air concentrations, with details as follows:

(1) $$\log\left(\lambda_{t}\right)=\beta_{1}X_{t}+\beta_{2}T_{t}+\beta_{3}H_{t}$$

where *λ*_*t*_ is the daily count of hospitalized T2DM patients with comorbidities on day *t*, *X*_*t*_ is the air pollutant concentration (e.g., NO_2_, PM_10_) on day *t* (unit: μg/m^3^), *T*_*t*_ is the daily average temperature (°C) on day *t*, and *H*_*t*_ is the daily relative humidity (%) on day *t*.

The relative risk increase (RRI) in HAs for T2DM per 10 μg/m^3^ increase of air levels were calculated by (RR‐1), with details as follows:

(2) $$\text{RRI}\%=\left(\exp\left(\beta_{1}*10\right)-1\right)*100\%$$

where *β*_1_ is the exposure‐response coefficient, representing the increase of daily hospitalizations linked to the 1 μg/m^3^ increase of air pollutants.

The short‐term association between air pollution and HAs for T2DM and T2DM with comorbidities were confirmed using single‐pollutant models. To distinguish the temporal effects of various lag structures, the single day (from the current day to 7 days before: from lag 0 to lag 7) and multi‐day moving average day (lag 01 to lag 07) were involved. Subgroups analyses were also conducted to estimate the effects of stratified modification by gender (male and female) and age (<45 years, and ≥45 years). The statistic differences from stratified analyses were confirmed by *Z*‐test (Evans et al., 2013).

All analyses were conducted using R (version 4.2.1). The *amapGeocode* package was adopted to geocode the home addresses. The *mgcv* package was involved in applying quasi‐Poisson regression. The Bonferroni correction was applied to correct the CI in stratified analysis and Benjamini‐Hochberg correction of *p*‐value was involved to balance false positive and false negative rates.

#### 2.4.2 Calculating the Number and Cost of T2DM Hospitalizations Due To Air Pollution

When a significant effect of air pollutant on T2DM hospitalizations estimated in the single‐pollutant model, it would be selected to estimate the attributable hospitalization number and cost, with World Health Organization's (WHO) air quality guidelines setting as reference levels (24 hr average: 25 μg/m^3^ for NO_2_, 45 μg/m^3^ for PM_10_, 15 μg/m^3^ for PM_2.5_, and 40 μg/m^3^ for SO_2_ (WHO, 2021)). Since the concentrations of SO_2_ were often below air guidelines and when the exposure‐response coefficient was minus, the attributable hospitalization number and costs of HAs for T2DM with comorbidities under these two situations would be excluded, owing to unavailability (Wu et al., 2020). To confirm the attributable exposure to exceeding air guidelines, the largest effect was adopted. The formulas are as follows:

(3) $$\text{AN}i=\left(\exp\left(\beta *\left(x_{i}-x_{0}\right)\right)-1\right)/\exp\left(\beta *\left(x_{i}-x_{0}\right)\right)*Ni$$

(4) $${\text{AC}}_{y\text{total}}={\text{AN}}_{y}*{\text{Cost}}_{y\text{total}}*{\text{CPI}}_{y}$$

(5) $${\text{AC}}_{y\text{pocket}}={\text{AN}}_{y}*{\text{Cost}}_{y\text{pocket}}*{\text{CPI}}_{y}$$

where AN*i* is the number of hospitalizations attributable to exceeding reference levels on day *i*; *x*_*i*_ (μg/m) is the observed level of air pollution on day *i*; *x*_0_ is reference level from air quality guideline of WHO, which is as the theoretical minimum threshold levels, when air levels below it posing no effect on HAs for T2DM with comorbidities (Evans et al., 2013); *Ni* is the number of HAs for T2DM with comorbidities on day *i*; AN is the sum of overall AN*i* during 2017–2019; ${\text{AC}}_{y\text{total}}$ and ${\text{AC}}_{y\text{pocket}}$ are the total hospital admission costs and out‐of‐pocket costs being attributable to exceeding air reference levels in year *y*; ${\text{AN}}_{y}$ is the sum of overall ${\text{AN}}_{y}$ during year *y* obtained from Equation 2; ${\text{Cost}}_{y\text{total}}$ and ${\text{Cost}}_{y\text{pocket}}$ indicate the case‐average total hospital admission expenses and out‐of‐pocket expenses in year *y*; ${\text{CPI}}_{y}$ is the Consumer Price Indices (CPI) from year *y* + 1 to 2019; ${\text{AC}}_{\text{total}}$ is the sum of ${\text{AC}}_{y\text{total}}$, and ${\text{AC}}_{y\text{pocket}}$ is the sum of ${\text{AC}}_{y\text{pocket}}$ during the study period. All main inferences were based on the pre‐specified lag window (lags 0–7 and lag 01–07), rather than on a single selected lag day. The peak lag estimate within this window was used only for the attributable‐burden calculation as a summary measure. The 95% confidence intervals for both ${\text{AC}}_{y\text{total}}$, and ${\text{AC}}_{y\text{pocket}}$ were derived by propagating the 95% confidence intervals of the estimated attributable hospitalization numbers, assuming the average cost per hospitalization for each condition as a fixed multiplier. This study utilizes case‐average costs to estimate the population‐level economic burden, which may not capture the full extent of heterogeneity in costs across different hospitals or individual patients. Future research with more granular, patient‐level data could provide further insight into the distributional effects of these costs. It is important to note that while our study uses a case‐average cost to estimate the population‐level economic burden, this approach may not capture the full extent of heterogeneity in costs across different hospitals and individual patient cases due to variations in clinical features and treatment decisions.

### 2.5 Sensitivity Analysis

To verify the robustness of the model, sensitivity analysis was involved. When a significant association found between one air pollutant and T2DM with comorbidities hospitalizations, a two‐pollutant model was introduced to test the stability of relationship after adding another pollutant. To minimize potential multicollinearity in two‐pollutant models, we systematically excluded pollutants with high pairwise correlations with the target pollutant, and included only those with relatively lower correlations as second pollutants.

## 3 Results

### 3.1 Data Description

The daily hospitalization number and costs for T2DM patients and T2DM patients with CVD, hypertension and PVD were listed in Table 1. During the study period, 92,381 T2DM HAs were recorded, of which 6,855 patients with CVD, 23,403 with hypertension, and 21,207 with PVD, with the average of 69, 6, 21, and 19 HAs per day, respectively. As for T2DM patients and T2DM patients with CVD, hypertension and PVD, male and female hospitalizations accounted for 52.3% and 47.7%, 45.3% and 54.7%, 49.3% and 50.7%, and 52.5% and 47.5%, separately. Obviously, HAs for patients ≥45 comprised over at least 91.1% not only in the T2DM patients but also including T2DM patients with CVD, hypertension and PVD. The average total expenses and out‐of‐pocket costs were 1.0 and 0.4, 1.0 and 0.3, 1 and 0.3, and 1.1 and 0.4 10 thousand CNY for T2DM patients and T2DM patients with CVD, hypertension and PVD, separately. The details for air pollutants and meteorological factors can be found in the previous study (Jiang et al., 2023).

**Table 1:** Descriptive Statistics of Daily T2DM Patients and T2DM Patients With Comorbidities in Sichuan Province During 2017–2019

Characteristics	Total	Descriptive statistics for daily data					
		Mean ± SD	Min	Max	*P*_25_	*P*_50_	*P*_75_
T2DM (*n*)	92,381	69 ± 48	1	202	20	68	108
Male	48,340	44 ± 19	1	113	29	43	58
Female	44,041	40 ± 17	1	102	27	39	52
<45	8,215	8 ± 4	1	22	5	7	10
≥45	84,165	77 ± 33	3	190	52	75	101
Total expenses^a^	935.8	1 ± 1.3	0	140.3	0.5	0.8	1.2
Out‐of‐pocket cost^a^	324.9	0.4 ± 0.7	1	81.8	0.1	0.2	0.4
T2DM with CVD (*n*)	6,855	6 ± 4	1	22	4	6	9
Male	3,105	3 ± 2	1	10	2	3	4
Female	3,750	4 ± 2	1	14	2	3	5
<45	146	1 ± 0	1	3	1	1	1
≥45	6,709	6 ± 4	1	21	4	6	8
Total expenses^a^		1 ± 1.1	0	19.3	0.5	0.8	1.1
Out‐of‐pocket cost^a^		0.3 ± 0.5	0	19.3	0.1	0.2	0.4
T2DM with hypertension (*n*)	23,403	21 ± 10	1	58	13	20	29
Male	11,534	11 ± 6	1	36	6	10	14
Female	11,869	11 ± 6	1	33	7	10	15
<45	1,028	2 ± 1	1	5	1	1	2
≥45	22,375	20 ± 10	1	57	13	20	27
Total expenses^a^		1 ± 1	0	54.8	0.5	0.8	1.1
Out‐of‐pocket cost^a^		0.3 ± 0.5	0	20.7	0.1	0.2	0.4
T2DM with PVD (*n*)	21,207	19 ± 10	1	48	12	19	26
Male	11,134	10 ± 5	1	28	6	10	14
Female	10,073	9 ± 5	1	29	5	9	12
<45	1,141	2 ± 1	1	7	1	1	2
≥45	20,066	18 ± 9	1	48	11	18	25
Total expenses^a^		1.1 ± 1.2	0	56.8	0.6	0.9	1.2
Out‐of‐pocket cost^a^		0.4 ± 0.5	0	13.5	0.1	0.2	0.4

^a^ Unit: case‐average expenses, 10 thousand CNY.

### 3.2 Health Effects of Air Pollution Exposure in Overall and Subgroup Population

**Figure 2:**
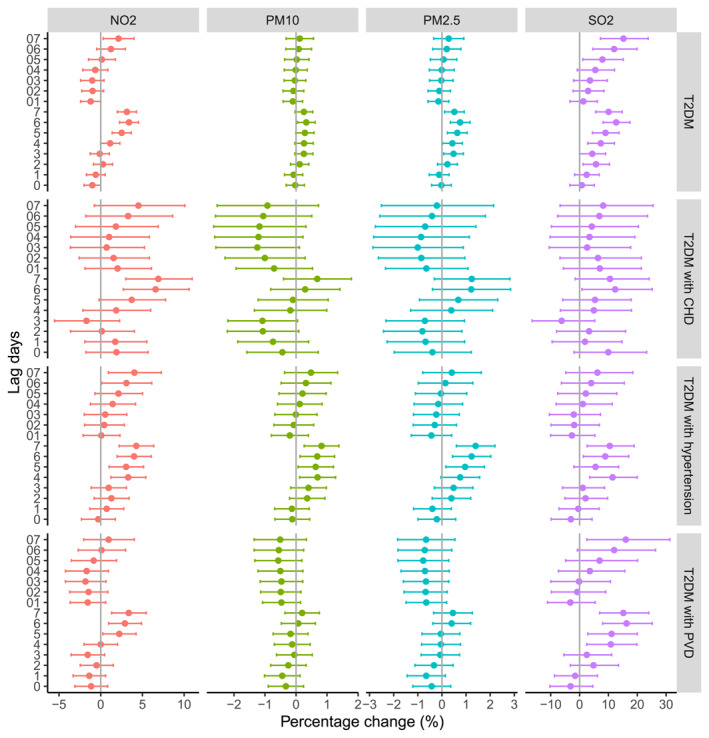
Percentage changes (95% confidence interval) in hospital admissions for T2DM, and T2DM with CVD, hypertension, and peripheral vascular disease were associated with a 10 μg/m^3^ increase in NO_2_, PM_10_, PM_2.5_, and SO_2_ on single day lags (from current day to 7 days before: lag 0–lag 7) and multi‐day moving average lags (from lag 01 to lag 07) using single pollutant models.

The associations of air pollutants increasing per 10 μg/m^3^ with HAs for T2DM patients and T2DM patients with CVD, hypertension and PVD in single pollutant models along different lag structures were shown in Figure 2. For T2DM, the effects of NO_2_, PM_10_ and PM_2.5_ reached their largest at lag 6 with RRI of 3.39% (95% CI: 1.87%, 4.93%), 0.33% (95% CI: −0.06%, 0.72%) and 0.76% (95% CI: 0.21%, 1.30%), while SO_2_ presented max RRI at lag 07 with value of 15.21% (95% CI: 4.37%, 26.65%). For T2DM with CVD, the largest effect of NO_2_ and PM_10_ can be found at lag 7 with RRI of 1.44% (95% CI: −1.04%, 3.95%) and 0.29% (95% CI: −1.20%, 1.80%), while PM_2.5_ and SO_2_ at lag 6 with RRI of 1.24% (95% CI: −0.86%, 3.36%) and 10.54% (95% CI: −5.97%, 28.53%). As for T2DM with hypertension, the largest effect of NO_2_, PM_10_, and PM_2.5_ at lag 7 with RRI were 4.27% (95% CI: 1.49%, 7.08%), 0.82% (95% CI: 0.07%, 1.58%) and 1.40% (95 CI%: 0.32%, 2.48%), while SO_2_ at lag 4 with RRI of and 11.44% (95% CI: 0.65%, 22.83%). For T2DM with PVD, the largest effect of NO_2_, PM_10_ and PM_2.5_ were at lag 7 with RRI of 3.36% (95 CI%: 0.59%, 6.17%), 0.20% (95 CI%: −0.55%, 0.95%) and 0.45% (95 CI%: −0.62%, 1.54%), while SO_2_ at lag 6 with RRI of 16.29% (95 CI%: 5.06%, 28.16%).

**Figure 3:**
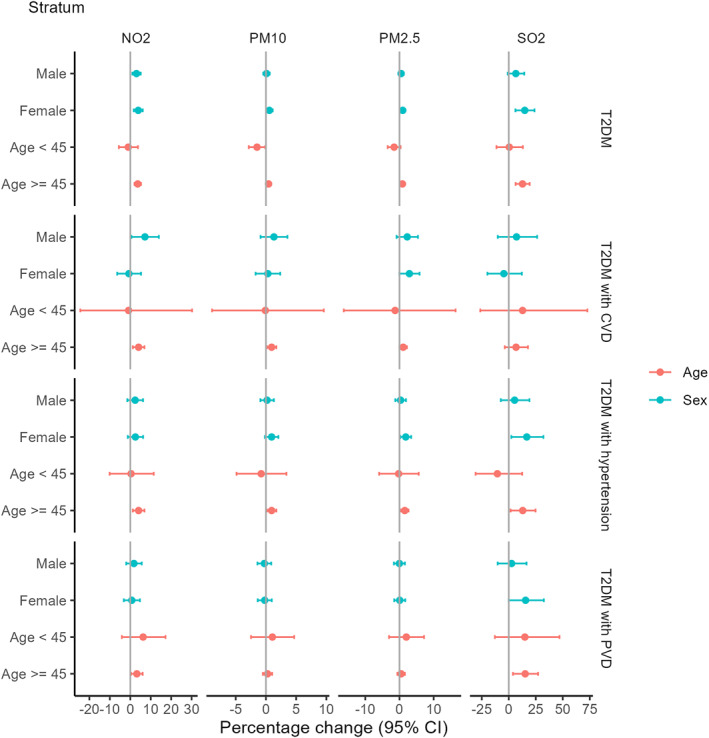
Percentage changes (95% confidence interval) of sex and age group in overall hospital admissions for T2DM, and T2DM with CVD, hypertension, and peripheral vascular disease were associated with a 10 μg/m^3^ increase in NO_2_, PM_10_, PM_2.5_, and SO_2_ in Sichuan Province in 2017–2019.

The results of stratified analyses by sex and age between air pollution and T2DM, and T2DM with CVD, hypertension, and PVD HAs were displayed in Figure 3, based on the results of the single‐pollutant model with the largest effects. For T2DM patients, NO_2_, PM_10_, PM_2.5_ are at lag 6, and SO_2_ is at lag 07. For T2DM patients with CVD, NO_2_ and PM_10_ are at lag 7, while PM_2.5_ and SO_2_ are at lag 6. For T2DM patients with hypertension, NO_2_ and PM_10_ and PM_2.5_ are at lag 7, while SO_2_ is at lag 4. For T2DM patients with PVD, NO_2_, PM_10_ and PM_2.5_ are at lag 7, while SO_2_ is at lag 6. The stratification results distributed not equally among subgroups. As for gender stratification, the effects of air pollutants. In age stratification, a significant effect of all four pollutants (NO_2_, PM_10_, PM_2.5_, and SO_2_) was observed on T2DM patients in older group (≥45 years), except for PM_10_ for patients with T2DM and CVD, and both PM_10_ and PM_2.5_ for those with T2DM and PVD.

The attributable number of HAs for T2DM with comorbidities and the associated hospitalization expense in Sichuan Province during 2017–2019 were displayed in Table 2, which were linked when exceeding WHO air quality standard. For total attributable cases linked to air pollution exceeding reference levels, the T2DM‐only patient group was 1,170 cases (95% CI: 180, 2,160) from NO_2_, 427 cases (95% CI: −307, 1,936) from PM_10_, and 1,234 cases (95% CI: 266, 2,203) from PM_2_._5_. For patients with T2DM and CVD, the attributable cases were 229 (95% CI: 57, 341) for NO_2_, 94 (95% CI: −107, 295) for PM_10_, and 209 (95% CI: −171, 590) for PM_2_._5_. Patients with T2DM and hypertension had 398 cases (95% CI: 142, 653) from NO_2_ and 356 cases (95% CI: 30, 682) from PM_10_. Finally, for the T2DM with PVD group, there were 311 attributable cases (95% CI: 56, 567) from NO_2_ exposure. For total expenses, the attributable costs linked with air pollution exposure were stratified by pollutant and comorbidity group. For T2DM‐only patients, the attributable costs were 38.40 (95% CI: 5.89, 70.91) for NO_2_, 14.01 (95% CI: −10.09, 63.56) for PM_10_, and 40.52 (95% CI: 8.73, 72.31) for PM_2_._5_. For patients with T2DM and CVD, the costs were 7.43 (95% CI: 1.85, 11.01), 3.04 (95% CI: −3.47, 9.55), and 6.79 (95% CI: −5.56, 19.14) for NO_2_, PM_10_, and PM_2.5_ respectively. Patients with T2DM and hypertension incurred costs of 12.17 (95% CI: 4.36, 19.98) due to NO_2_ and 10.89 (95% CI: 0.91, 20.86) due to PM_10_. For the T2DM with PVD group, the attributable costs were 10.82 (95% CI: 1.94, 19.71) for NO_2_.

**Table 2:** The Attributable Hospitalization Number and Corresponding Costs for T2DM and T2DM With Comorbidities Due To Exceeding Air Pollution Exposure Using WHO Air Quality Standard in Sichuan Province, 2017–2019

Category	Lag days	AN	AC_total_ ^a^	AC_pocket_ ^a^
T2DM				
NO_2_	lag 6	1,170 (180, 2160)	38.40 (5.89, 70.91)	15.72 (2.41, 29.02)
PM_10_	lag 6	427 (−307, 1,936)	14.01 (−10.09,63.56)	5.73 (−4.13, 26.02)
PM_2.5_	lag 6	1,234 (266, 2203)	40.52 (8.73, 72.31)	16.59 (3.58, 29.60)
T2DM with CVD				
NO_2_	lag 7	229 (57, 341)	7.43 (1.85, 11.01)	2.33 (0.58, 3.45)
PM_10_	lag 7	94 (−107, 295)	3.04 (−3.47, 9.55)	0.95 (−1.09, 2.99)
PM_2.5_	lag 6	209 (−171, 590)	6.79 (−5.56, 19.14)	2.13 (−1.74, 6.00)
T2DM with hypertension				
NO_2_	lag 7	398 (142, 653)	12.17 (4.36, 19.98)	3.99 (1.43, 6.55)
PM_10_	lag 7	356 (30, 682)	10.89 (0.91, 20.86)	3.57 (0.30, 6.84)
T2DM with PVD				
NO_2_	lag 7	311 (56, 567)	10.82 (1.94, 19.71)	3.21 (0.57, 5.84)

*Note.* AN, AC_total_, AC_pocket_ were calculated based on the largest effect estimates in single pollutant models.

^a^ Unit: million CNY. The annual costs were measured at the CPI from 2019. The 95% confidence intervals for costs were calculated based on the confidence intervals of the attributable hospitalization numbers. A negative lower bound indicates that the estimated cost is not statistically significant at the 95% confidence level, meaning the effect on hospitalizations cannot be distinguished from zero.

### 3.3 Sensitivity Analysis

To estimate the coupled effects of other pollutants on the HAs for T2DM, the results of the two‐pollutant model were displayed in Table 3. Based on the largest effect from single‐pollutant models, T2DM and T2DM patients with CVD is lag 6, T2DM patients with hypertension is lag 7 for NO_2_, PM_10_, PM_2.5_ and SO_2_, while T2DM patients is lag 7 for NO_2_, PM_10_ and PM_2.5_ with lag 6 for SO_2_. When involving the second pollutant, the effect of two‐pollutant model would vary to some extent compared to the result of single‐pollutant model. Some would be weakened, while others would be enhanced. Like, for T2DM patients with CVD the effect of PM_10_ and PM_2.5_ were stronger after adjusting second pollutant, while the impact of SO_2_ would be smaller after introducing second pollutant.

**Table 3:** Percentage Changes (%) and Its 95% CI of HAs for T2DM With Comorbidities Were Associated With 10 μg/m^3^ Increase of Air Pollutants in Two‐Pollutant Models

Pollutants^a^		T2DM	T2DM with CVD	T2DM with hypertension	T2DM with PVD
NO_2_	–	3.39 (2.26, 4.54)	6.58 (2.71, 10.60)	4.27 (2.21, 6.36)	3.36 (1.30, 5.45)
	+ PM_10_	5.17 (3.51, 6.86)	−2.03 (−8.08, 4.43)	2.03 (−3.77, 8.17)	0.82 (−4.76, 6.72)
	+ PM_2.5_	3.97 (2.32, 5.65)	−2.83 (−8.56, 3.27)	2.71 (−2.96, 8.71)	−1.06 (−6.40, 4.57)
	+ SO_2_	2.17 (0.53, 3.85)	0.50 (−4.60, 5.88)	3.85 (−0.86, 8.79)	−3.11 (−7.40, 1.38)
PM_10_	–	0.33 (0.04, 0.62)	0.29 (−0.81, 1.42)	0.82 (0.26, 1.38)	0.20 (−0.36, 0.75)
	+ NO_2_	−0.62 (−1.03, −0.20)	1.65 (−0.78, 4.15)	0.74 (−1.54, 3.08)	−2.76 (−4.85, −0.63)
	+ SO_2_	−0.28 (−0.64, 0.08)	1.26 (−0.79, 3.34)	1.56 (−0.30, 3.46)	−2.35 (−4.04, −0.62)
PM_2.5_	–	0.76 (0.35, 1.16)	1.23 (0.44, 2.03)	1.40 (0.60, 2.20)	0.45 (−0.35, 1.26)
	+ NO_2_	−0.28 (−0.64, 0.08)	3.47 (0.07, 6.98)	0.48 (−2.57, 3.63)	−2.38 (−5.22, 0.55)
	+ SO_2_	−0.28 (−0.64, 0.08)	2.81 (−0.18, 5.89)	1.79 (−0.79, 4.44)	−2.32 (−4.69, 0.11)
SO_2_	–	12.68 (8.14, 17.42)	12.34 (0.81, 25.19)	10.47 (2.61, 18.92)	16.29 (8.03, 25.19)
	+ NO_2_	6.22 (−0.04, 12.88)	0.72 (−10.84, 13.78)	−6.48 (−16.96, 5.33)	−1.32 (−11.28, 9.76)
	+ PM_10_	15.48 (9.64, 21.63)	−1.50 (−13.11, 11.66)	−7.10 (−17.67, 4.82)	0.29 (−9.94, 11.68)
	+ PM_2.5_	12.54 (8.14, 17.42)	−2.95 (−14.4, 10.09)	−6.72 (−17.36, 5.28)	0.26 (−10.01, 11.71)

^a^ The peak‐lag estimate from the single‐pollutant model is selected with second pollutant included for adjustment.

## 4 Discussion

In this study, a time‐stratified case‐crossover analysis is performed to estimate the associations between air pollutant exposure and risks of HAs for T2DM patients and T2DM patients with comorbidities in Sichuan Province. As presented, the short‐term exposure to air pollution was associated with the hospitalization risks of T2DM with CVD, hypertension and PVD. As for stratified analysis, the older group were exposed to higher effects of air pollution. Besides, the attributable number and corresponding hospitalization costs for T2DM patients and T2DM patients with comorbidities due to exceeding air pollution exposure were estimated.

Our findings are consistent with prior evidence suggesting that air pollution negatively impacts the health of T2DM patients, particularly those with comorbidities. NO_2_ exposure, in particular, exhibited the most pronounced short‐term effects across all patient groups. For T2DM patients without specific comorbidities, the maximum RRI was 3.39% (95% CI: 1.87%, 4.93%) observed at lag 6. For patients with comorbidities, the largest effects typically occurred at lag 7. And the RRIs for those with CVD, hypertension, and PVD were 1.44% (95% CI: −1.04%, 3.95%), 4.27% (95% CI: 1.49%, 7.08%), and 3.36% (95% CI: 0.59%, 6.17%), respectively. This temporal pattern suggests a delayed physiological response to gaseous pollutants in diabetic individuals with vascular complications (Su et al., 2020).

Previous studies from other areas found that high air pollution were linked with the increase outpatient visits and hospitalizations for T2DM patients with comorbidities in Shandong Province of China and São Paulo of Brazil, which mainly focused on T2DM patients with CVD (Pereira Filho et al., 2008; J. Zhang et al., 2022). A study in America found that many diabetic people with cardiovascular as secondary complications, as a group they show larger effect to PM than do people with cardiovascular disease and no diabetes (Zanobetti & Schwartz, 2001). These data indicate that individuals with diabetes who also have cardiovascular disease may be susceptible to the short‐term effects of air pollution. One possibility is that the added response may be due to people with diabetes associated with endothelial dysfunction. When vascular endothelial cells in these individuals are compromised, they may be more susceptible to the effects of air pollutants. Besides, diabetic patients are linked with disproportional reactive oxygen species formation, which would increase tissue damage (Maritim et al., 2003).

In a study of the short‐term effects between air pollution and blood lipid markers levels in Wuhan, China reported that higher levels of blood apolipoprotein B and lipoprotein a in elderly T2DM patients with hypertension (Xiao et al., 2016). Exposure to air pollution has an influence on blood pressure, as four meta‐analysis reported that increasing per 10 μg/m^3^ PM_2.5_ is consistently associated with 1–3 mm Hg elevations in diastolic blood pressure over the ensuing few days (Rajagopalan et al., 2018). The plausible mechanisms by which air pollutants affect the development of T2DM with hypertension include the production of systemic inflammation, oxidative stress and initiation of autonomic nervous system imbalance (Brook et al., 2004). Oxidative stress as a notable role in genesis of insulin resistance could be induced by air pollution (Kelly, 2003). In addition, autonomic nervous system imbalance can promote constriction, which has been found after exposure to air pollution, introducing hypertension and decreasing insulin sensitivity (Lindmark et al., 2003).

Furthermore, few existing studies have explored the short‐term effect of air pollution on HAs for T2DM patients with PVD, which needs more studies to further explore relationships. But there is a study found that long‐term air pollution exposure would have adverse effects on the development of PVD (Brook, 2007). A study in China collected 37 PVD‐related parameters reported that diabetes and hypertension being the third and second leading cause of PVD. Diabetic patients are about 1.71 times more likely to develop as PVD than nondiabetic patients (Song et al., 2019).

In stratified analyses, the associations between ambient air levels with HAs for T2DM patients with comorbidities distributed not equally among subgroups. In age stratification, air pollution generally had significant impacts on patients with age ≥45 years, which was consistent with previous studies (Li et al., 2019). The elderly are facing with a certain degree of degradation in different bodily functions, reducing them being more susceptible to air pollution. And the young‐aged groups have better physical fitness, so less effects of air pollution would be found on them. Besides, the elderly groups spend more time in outdoors than the young groups who is with work necessities (Tong et al., 2015). In gender analysis, females were associated with higher exposure to air pollution for T2DM patients with hypertension and PVD, while male patients of T2DM with CVD exposure to NO_2_ involving with higher risks. Female are more susceptive to air pollution for T2DM with PVD group, which was in line with previous studies (Hsieh et al., 2009). As for the gender difference, it is derived from biological and non‐biological factors. In view of sex‐specific differences in biologic susceptibility, this may related to true differences in biologic susceptibility, as observations from the Women's Health Study which reported that a greater susceptibility of women to air pollution‐mediated cardiovascular events (Miller et al., 2007). In contrast, the sex prediction may be related to exposure assessment error, especially for male who is likely to spend more time in outdoors compared with females. Besides, different life stress and socio‐economic status were involved for females and males (Seeman et al., 2002).

In this study, the hospitalization costs linked with HAs for T2DM patients with comorbidities to air pollutants were estimated, implying considerable importance for improving air quality and preventing the development of diabetic comorbidities. Taking the WHO air concentrations as references, the highest attributable hospitalization HAs and costs for T2DM patients with comorbidities were associated mostly with NO_2_ then followed by PM_2.5_ and PM_10_. A previous study concerning the associations of incident T2DM with air pollutants found that traffic‐related pollutants, like NO_2_, were found with stronger effect than particulate matter (Krämer et al., 2010). NO_2_ was a pollutant primarily from traffic, the results may indicate that traffic‐related pollutants were linking strongly with the increased incidence of T2DM. When compared with PM_10_, PM_2.5_ with a smaller diameter of particles and higher surface‐to‐volume ratio, these make it easier to cross the blood‐brain barrier carrying with more absorbed toxic substance, resulting in neuro‐inflammation, neuronal signaling dysfunction and immune responses and even damage to neurological system (Jia et al., 2018). To date, this is the first study which has estimated the hospitalization costs of HAs for T2DM patients with comorbidities due to air pollution, thus studies of this field would be valuable for policy making and societal perspective. In terms of evidence from this study, it is necessary to control the levels of air pollution, which could reduce the hospitalization costs.

The characteristics of this study are significant. First, this is the first investigation on the associations between air pollution and HAs for T2DM patients with comorbidities in Sichuan. T2DM as a typical chronic disease always involves various comorbidities, leading to heavy hospitalization costs to family and society. Second, the hospitalization costs and attributable hospitalizations owing to exposure to air pollutants have been calculated, which could provide evidence for prevention of T2DM patients with commodities. Third, 9 cities/prefectures of Sichuan Province and sizable records of T2DM patients have been involved, which could reflect the overall figure in Sichuan province. And there are also some limitations for this study. First, limited by the data availability, not all potential confounders were involved, like indoor air pollution and time spent outdoors. Then, the hospitalization costs estimated in this study tended to be understated, since indirect costs and outpatient expenditures were involved in real status, which are not analyzed. Third, air pollutants are always made up of different components, varying composition under the influence of emission sources and prevailing weather conditions. But only the overall impacts of air pollution were involved.

## 5 Conclusions

This study demonstrated the short‐term exposure to air pollution (NO_2_, PM_10_, PM_2.5_, and SO_2_) was linked with increasing HAs for T2DM patients with comorbidities in Sichuan Province, indicating the necessity for strategies on public health promotion based on air pollution. Besides, the hospitalization costs of T2DM patients with comorbidities owing to air pollution exposure were estimated. It is strongly suggested that further studies concerning epidemiology and comorbidities perspective should be carried out to verify the causal relationship between air pollutants and T2DM patients with comorbidities.

## Conflict of Interest

The authors declare no conflicts of interest relevant to this study.

## Data Availability

The raw/processed patient data involve individual information, which are not accessible to the public due to Medical Ethics Committee of the Affiliated Hospital of Chengdu University of Traditional Chinese Medicine's data policy. All analyses in this study are made with software R version 4.2.1, licenses and other information can be found at https://www.R‐project.org (R Core Team, 2022).
